# Adaptive sparse sampling for quasiparticle interference imaging

**DOI:** 10.1016/j.mex.2022.101784

**Published:** 2022-07-13

**Authors:** Jens Oppliger, Berk Zengin, Danyang Liu, Kevin Hauser, Catherine Witteveen, Fabian von Rohr, Fabian Donat Natterer

**Affiliations:** a Department of Physics, University of Zurich, Winterthurerstrasse 190, Zurich CH-8057, Switzerland; b Department of Physics, Harvard University, 17 Oxford Street Cambridge, MA 02138, United States of America; c Department of Quantum Matter Physics, University of Geneva, 24 Quai Ernest-Ansermet, Geneva CH-1211, Switzerland

**Keywords:** Sparse Sampling, Quasiparticle interference imaging, Fourier Transform scanning tunneling microscopy, Quantum materials characterization

## Abstract

Quasiparticle interference imaging (QPI) offers insight into the band structure of quantum materials from the Fourier transform of local density of states (LDOS) maps. Their acquisition with a scanning tunneling microscope is traditionally tedious due to the large number of required measurements that may take several days to complete. The recent demonstration of sparse sampling for QPI imaging showed how the effective measurement time could be fundamentally reduced by only sampling a small and random subset of the total LDOS. However, the amount of required sub-sampling to faithfully recover the QPI image remained a recurring question. Here we introduce an adaptive sparse sampling (ASS) approach in which we gradually accumulate sparsely sampled LDOS measurements until a desired quality level is achieved via compressive sensing recovery. The iteratively measured random subset of the LDOS can be interleaved with regular topographic images that are used for image registry and drift correction. These reference topographies also allow to resume interrupted measurements to further improve the QPI quality. Our ASS approach is a convenient extension to quasiparticle interference imaging that should remove further hesitation in the implementation of sparse sampling mapping schemes.

• Accumulative sampling for unknown degree of sparsity

• Controllably interrupt and resume QPI measurements

• Scattering wave conserving background subtractions

## Graphical abstract

**Figure:**
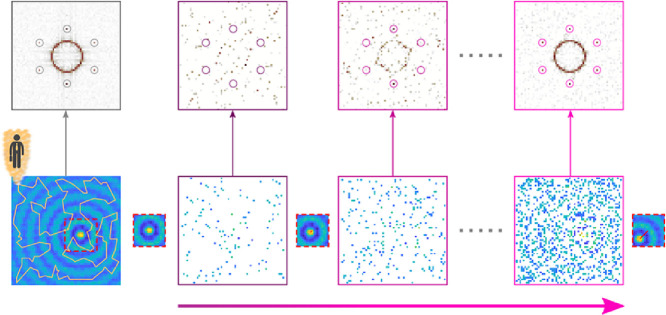

**SPECIFICATIONS TABLE**

**Table:** 

Subject Area;	Physics and Astronomy
More specific subject area;	*Condensed Matter Physics*
Method name;	*Adaptive Sparse Sampling for Quasiparticle Interference Imaging*
Name and reference of original method;	*Sparse sampling for fast quasiparticle-interference mapping, Jens Oppliger and Fabian Donat Natterer, Physical Review Research****2****, 023117 (2020),*
Resource availability;	*N.A.*

## Broader context

Sparse sampling for quasiparticle interference (QPI) imaging is a novel scanning tunneling microscopy mapping scheme that promises fundamentally shorter measurement times than conventional grid spectroscopy [1,2]. The speed-up from sparse sampling comes from measuring far fewer samples than suggested by the Nyquist-Shannon sampling theorem. The condition for a successful compressed sensing^1^ recovery [3,4] is a high degree of signal sparsity in some representation space. For QPI imaging, the Fourier transform of the local density of states (LDOS) can be highly sparse [1,5], which incidentally is also the representation space. When sparse sampling is paired with faster point-spectroscopy [6], QPI mapping becomes orders of magnitude faster than conventional grid methods [2]. This leaves the question as to the required sub-sampling as an unresolved practical issue because the mathematical prescriptions that determine successful recovery [7] depend on *a-priori* insight about the signal-to-noise level and the initially unknown degree of signal sparsity.

Here we introduce an adaptive sparse sampling (ASS) approach through which we iteratively increase the amount of sub-sampling during runtime to accumulate LDOS measurements until a satisfactory signal quality becomes visible in the preview snapshots of the recovered QPI images. Our ASS procedure further enables an interruption of QPI imaging at recurring exit points from which the mapping can be resumed to increase the cumulative amount of LDOS measurements. The interleaving of regular topographic scans with the sparsely sampled LDOS measurements enables image registry and drift correction that may be required due to thermal drift or after repositioning of the tip in the aftermath of longer interruptions, such as the refilling of one's cryostat. Through ASS, an experimenter can design open-ended mapping tasks and tackle the QPI inspection of systems for which there is only incomplete information available about the sparsity level and noise. Our ASS method allows to monitor the signal quality in-operando for feedback of ongoing measurements and to stop/interrupt measurements at opportune moments. We emphasize here that the number of required ASS iterations will, aside from the signal to noise ratio, depend on the degree of sparsity (number of unique momenta) but not on complexity (relationship between momenta) of the QPI information, which some of us discussed earlier for QPI [1] and which is rigorously stated in general terms for compressive sensing [8].

## Method detail

**Fig. 1:**
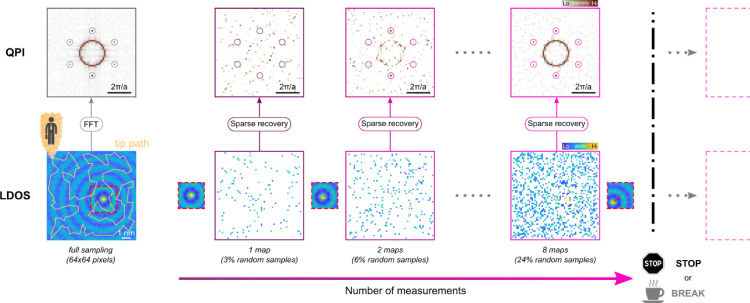
Adaptive sparse sampling concept. The top panels show the quasiparticle interference (QPI) patterns of a simulated surface state. The leftmost QPI pattern is the ground truth that we want to adaptively reconstruct from an accumulation of sparsely sampled local density of states (LDOS) measurements. The reconstruction gets gradually better with increasing number of cumulative measurements. The bottom panels show the LDOS with the ground truth in the leftmost panel. The orange line indicates the traveling salesperson path that is used to obtain the sparsely sampled LDOS measurements in the adaptive sampling scheme. The small insets between the maps illustrate the usage of smaller LDOS or topographic maps that can be intersected between adaptive sampling measurements to align the individual measurements for the combined sparse recovery and the red line indicates the drift vector. In an actual measurement one should use regular topography scans due to the much shorter acquisition time. The ASS concept allows to pause, resume, or interrupt the measurements, for instance when the QPI pattern has a sufficient quality.

The working principle of our ASS approach is summarized in Fig. 1, where we first exemplify the concept using a simulated Shockley surface state consisting of a standing wave modulating the LDOS, atomic corrugation, and added Gaussian noise. The full LDOS and QPI, shown here to the left for one energy, is the desired information (ground truth) we seek to recover. The scattered surface state is modelled using a modified Bessel function of the first kind [9]. The random motion path overlay indicates the traveling salesperson route for one sparse sampling measurement and the dashed box marks the reference topography (or LDOS) that is used for image registry, as described below. To approach the QPI ground-truth of the surface state ring and the Bragg peaks in an adaptive measurement scheme by gradually accumulating LDOS measurements, we proceed according to the following four steps:

1 Path generation
2 Path combination
3 Mapping and preview
4 Postprocessing corrections.

1 **Path generation**

Prior to the actual measurement, we prepare the tip travel paths. From all possible locations on a grid of size N=n×n, we select *p*-times qN random locations with *q* ∈(0,1] being a small fraction. For every subset *p*, we calculate a near optimal solution to the traveling salesperson path (TSP) using a genetic [10] or cheapest-insertion [11] algorithm with fixed start and end coordinates. These two fixed points are shared among all paths to simplify their connection between the individual iterations, and they help build a catalogue of precalculated paths that can be quickly combined in the preparation of an actual measurement. Here we limit ourselves to a maximal number of qN=3000 locations per path because of the poor scaling of TSP calculations (computational NP-hardness). Note that the choice of these locations is compatible with informed sampling [1] in which we modify the probability of selecting a location based on various criteria, such as: proximity to the border, no-go areas, or defects of high LDOS scattering intensity. The average time for calculating one TSP path segment containing 3000 locations is about 2.5 hours per CPU core (AMD EPYC 7302P) using a genetic algorithm. The path generation using a cheapest insertion algorithm is faster but results in a slightly longer overall path length. We precalculated ∑p=100 TSP paths of which we typically use 20 to 30 in the present demonstration, equivalent to 60’000 to 90’000 locations at which the full LDOS is measured.

2 **Path combination**

The experiment starts by measuring a regular topography, preferably centered around topographic features that may serve as fiducial markers to aid image registry. We then proceed to set up the path for an ASS experiment where we define the number of TSP path subsets that we measure consecutively before we scan a regular topography. The reason for enchaining several TSP segments is the limited number of locations that we can afford to compute for one path and the relatively long duration of the topographic scans in our early ASS implementation, which consisted of measuring a full spectrum also for every location in the reference topography. Since full spectroscopic information as reference for the topography is not necessary, a regular topography scan would be sufficient and much faster. The end of the topography scan concludes the first ASS iteration. Since salient topographic features for image registry may be off-center, we generate a jittery random-walk path from the last location of the TSP segment to the start location of the topography scan and another jittery path from the last location of the topography scan to the start of the next TSP subset. This allows the system to settle creep that may arise from larger changes in the scan piezo voltage from repositioning the tip. By default, the ASS routine then proceeds to the next path subsets and records another topography scan at its end and so forth. The correlation between the reference-topographic scans yields the effective displacement that occurred from one iteration to the next due to thermal drift or residual creep. This displacement information is used at the end of the QPI mapping to accurately assign the correct coordinates to each location, which improves the overall reconstruction quality, as discussed further below.

3 **Mapping and preview**

We obtain the QPI information indirectly by first measuring the LDOS at every of the chosen locations, as described by the previously generated path combination, and then perform a sparse recovery of the QPI image by solving a basis pursuit denoising problem provided by the sparse least-squares solver SPGL1 [12,13] written in MATLAB. The procedure to recover the QPI pattern from LDOS measurements is equivalent to our previous sparse sampling implementations [1,2] and does not depend on the ASS method per se. The only difference is that our measurement matrix that feeds the sparse sampling recovery solver is growing with every iteration; see supplementary materials (*SM*) for a brief mathematical description. We measure the LDOS either via conventional bias spectroscopy or via parallel spectroscopy [2] for several energies. All LDOS measurements of one energy are treated independently to the LDOS at a different energy. In addition to the spectral information, we also record the time at which each spectrum is saved. This timestamp could later serve for displacement correction [1]. The series of panels in Fig. 1 shows how more and more LDOS measurements (bottom row) are accumulated after every TSP path iteration, leading to a growing measurement matrix. The top-row shows the QPI image that is obtained from the sparse recovery of the cumulative LDOS. We can appreciate how the quality of the recovered QPI pattern improves with more sampling. To use this mapping scheme in an actual measurement, we limit ourselves to the reconstruction of a few selected energies during the regular topography scans. The sparse recovery computation of the QPI pattern from a 1024×1024 grid for a single energy slice takes only a few minutes, leaving sufficient time to decide whether to proceed with the mapping or to stop. This preview option is useful to gauge the quality of the QPI mapping and to get a sense for the level of detail already present in the QPI maps.

4 **Postprocessing corrections**

**(a) Accounting for tip instabilities:** A frequent disturbance in STM investigations are slight and spontaneous tip-changes that manifest as an abrupt change in the conductance. To salvage such measurements, we perform a global background correction that conserves both the long-range LDOS modulations and the LDOS relationship between the measurement locations. Consequently, these background corrections preserve the spatial frequency content in the LDOS from which the QPI signature is obtained. We assume that the measured conductance spectra are a convolution of tip ($\varrho_{T}$) and sample ($\varrho_{S}$) density of states ($dI/dV\left(E\right)\propto \varrho_{S}\varrho_{T}$) [14]. If $\varrho_{T}$ changes, this will merely have an energy dependent multiplicative effect on the conductance. We also assume that we start our measurements or are able to identify segments with a well-behaved tip that has finite conductance in the relevant energy interval. The conductances measured for different energies/bias voltages are treated independently. In order to combine the LDOS data, measured for different TSP segments and with different tip density of states $\varrho_{T}$, we proceed as follows: We first calculate for every TSP segment s the respective mean $m_{s}=\sum_{k}\varrho_{S}^{k}\varrho_{T}^{k}/qN$ and standard deviation $\sigma_{s}=\sqrt{\sum_{k}{\left(\varrho_{S}^{k}\varrho_{T}^{k}-m_{s}\right)}^{2}/qN}$ independently for all bias voltages and locations *k* within that TSP segment. We then select one TSP segment as reference (preferably one without tip-changes, which typically is the first one) and subtract the individual mean from all other TSP segments, divide by their respective standard deviation, multiply by the standard deviation of the reference, and add the mean of the reference, $\varrho_{S}\varrho_{T}^{\prime }=\left(\varrho_{S}\varrho_{T}-m_{s}\right)\sigma_{ref}/\sigma_{s}+m_{ref}$. We apply this correction to TSP segments that show no tip-changes during their measurement, although such data could be handled in a similar way. One could identify the measurement index at which the tip-change occurred using an edge-detector and then treat one sub-segment as reference for the remaining sub-segments with the correction just described. Note that a different tip configuration might have a detrimental impact on the spatial frequency content due to its spatial convolution that inhibits the resolution of high-momenta states. Such states are however not relevant to our present demonstration.

**(b) Linear drift correction:** As mentioned, the interleaved topographic images serve to create spatial references for the TSP path segments such that the LDOS measurements of distinct ASS iterations can be combined into one measurement matrix for QPI reconstruction. Depending on the stability of the system or whether the measurement has been interrupted for some time, the image registry can become essential to ensure the proper spatial relationship between LDOS measurements, notably between those of distinct TSP subsets. The knowledge of the exact coordinates is what enables the sparse recovery for QPI imaging. To account for the time-dependence between the topography images and the different distances between consecutive locations, we can use the timestamp information $t_{k}$ of every location *k* that we have recorded alongside the LDOS (*SM*). Following our previous work [1], we first determine the global displacement vector $\vec{v\;}=\left(v_{x},\;v_{y}\right)$ from the image registry between reference topographies and the drift-speed $d\vec{v}/dt$, calculated from the total time that has passed in-between the reference images. We assume linear displacement and accordingly attribute to every location a proportional correction ${\vec{r_{i}}}^{\prime }=\vec{r_{i}}+\frac{d\vec{v}}{dt}\left(t_{k}-t_{k-1}\right)$. This adjusts the effective coordinates of the LDOS measurements, which helps in the QPI reconstruction because it improves the reliability of the spatial relationship in the LDOS modulations. In the (*SM*), we discuss nonlinear drift and scenarios where these assumptions cease to apply.

## Method validation

**Fig. 2:**
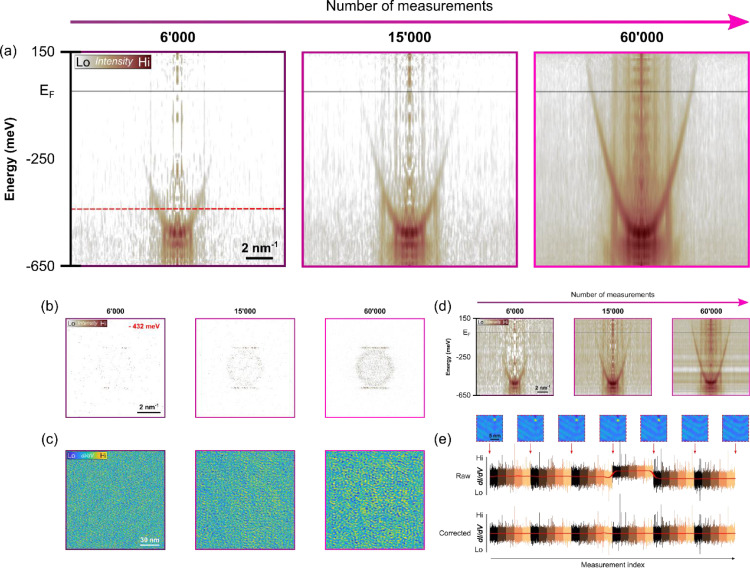
Validation of adaptive sparse sampling using Au(111). (a) Dispersion plots of Au(111), showing the parabolic dispersion of the nearly-free electron like Shockley surface state. A background correction, as described in the text, has been applied and the dispersion plots are created from azimuthal averages of QPI patterns, such as the ones shown in (b). The quality improves with increasing number of adaptively sampled local density of states (LDOS) measurements. **(b)** QPI patterns obtained after sparse recovery of adaptively sampled LDOS, showing the gradual improving quality with increasing number of measurements. **(c)** LDOS obtained from an inverse Fourier transform of the QPI patterns in (b). Every ASS increment consisted of 3’000 locations and a reference topography/LDOS was recorded after 5 such ASS increments. **(d)** Same dispersion plots as in (a) but without background correction applied, showing the detrimental effect of tip-changes. **(e)** Reference topographies/LDOS maps that are intersected between the adaptive sparse sampling loop (interleaving shown by vertical red arrows) to align the LDOS measurements for the cumulative sparse reconstruction. The two bottom rows show an example of the LDOS trace before and after the application of our background correction. (setpoint: V_b_ = -250 mV, V_drv_ = 400 mV, f_drv_ = 1600 Hz, I_t_ = 1.5 nA, t_spc_ = 20 ms, T = 4.3 K, grid size 1024 × 1024).

In order to validate the functioning for an actual measurement, we choose the well-known model system Au(111), which is characterized by a Shockley surface state and a $\left(22\;\times \sqrt{3}\right)$ herringbone reconstruction [15]. In our previous work [2], we have measured the dispersion relation of the nearly free electron gas using all combinations of conventional spectroscopy, parallel spectroscopy and sparse sampling for QPI imaging to ensure that our spectroscopies and mapping modes reproduce the Au(111) reference signatures. However, in those measurements we had measured all LDOS values in a single sweep, that is, not adaptively. We proceed here to reproduce these characteristic Au(111) signatures using the adaptive sparse sampling implementation. For the present ASS demonstration, one iteration consists of 5 random walk segments with 3’000 measurement locations each followed by one reference topography. The first iteration is started with an additional topography. Since a reference topography requires about 20 minutes, it can also be interpreted as an interruption/break of the sparse sampling mapping scheme. The panels (a)-(c) in Fig. 2 show the evolution of the QPI reconstruction with an ever-growing number of adaptively sampled LDOS values that are added to the reconstruction with the applied background correction mentioned previously in the post-processing section. From the dispersion plot in panel (a), one clearly notices how more band-structure details emerge with increasing number of measurements. We also see that the states closer to the reciprocal zone center appear at lower sampling than high momentum-transfer states, which is related to the effective level of sparsity at the respective energies in *q*-space. When the surface state has a higher *q*-value, it is represented by more wavevector values simply due to the larger circumference [1]. Similarly, the herringbone reconstruction is visible from a rather low sampling already, showing the dependence of the reconstruction efficiency with sparsity in *q*-space. We further elaborate on this in the discussion below. The inverse Fourier transforms of the QPI images are shown in panels (c) and reveal the spatially resolved LDOS with standing wave patterns and the surface reconstruction. For comparison, we also show the same data without background correction in panel (d). The dispersion plots are perturbed by sudden jumps that are caused by tip-changes, occurring during our measurements. This can also be seen in Fig. 2(e), where we show how the background correction accounts for and removes sudden changes in the conductance. As mentioned above, this correction is benign with regards to the spatial wavevector information. Although of lesser quality, the uncorrected QPI reconstruction does still show an improvement with increasing ASS iterations, which is crucial for quality assessment during runtime.

## Discussion und tweaks

**Fig. 3:**
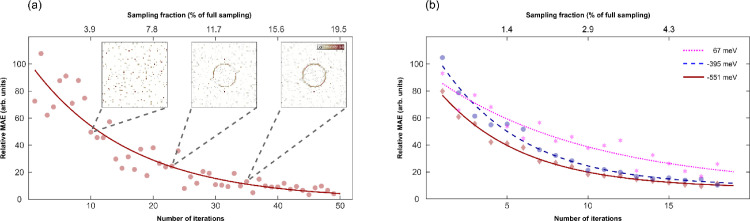
Quality of quasiparticle interference reconstruction with increasing sampling fraction. (a) The decreasing relative mean absolute error (MAE) (SM) between two consecutive ASS iterations shows how the quality of the QPI reconstruction rapidly increases with growing number of iterations, here demonstrated for the simulated surface state of Fig. 1 with known ground-truth. **(b)** The relative MAE for the actual QPI measurement on Au(111) shown in Fig. 2 for three different energies, follows a similar trend. Since the relative MAE can be calculated after every ASS iteration at runtime, it provides feedback on the status of the QPI data and enables an informed decision about how much more sampling would be required. The different slopes in the three curves reflect the reduced sparsity at higher energies that requires appropriately more sampling. Note that the data in both (a) and (b) follow a power law behavior as indicated by the fitted lines.

We have demonstrated the mechanism through which QPI measurements can be adaptively and sparsely sampled. We now turn to discussing how to generate accompanying quantitative feedback that could serve as a quality assessment for the QPI data. To that end, we calculate for every ASS step the relative mean absolute error (MAE) *(SM)* between the cumulative measurements with and without the latest iteration, which can be done at runtime. As shown Fig. 3(a) we notice a rapidly decreasing MAE with progressing ASS iterations, indicating the gradual approaching of the ground truth. The insets in (a) relate this quantitative measure to the more familiar QPI pattern for visual quality assessment. This indicates that the MAE could serve as a useful indicator for the QPI quality, enabling the numerical tracking of the QPI progress in automated decision-making approaches such as reinforcement learning. In panel (b), we apply the MAE evaluation also to our actual QPI measurement of the Au(111) surface state. The MAE likewise nicely tracks the quality of the QPI reconstruction. We further notice a dependence on the LDOS energy with regards to MAE improvement, which can be traced back to the varying degree of sparsity in the QPI pattern for different energies. This can be seen when comparing the MAE for -551, -395, and 67 meV. The momentum of the energetically more positive surface state is higher, which translates into a lower sparsity because the surface state consists of more values due to its larger circumference.

Up to this point, we have mostly assumed well-behaved systems with a handful of smaller tip-changes for which we have introduced a mitigating background correction. We obtain less reliable results when nonlinearities start to become prominent, for instance in nonlinear thermal drift, or nonlinear responses of the piezo-actuators to fast tip-speeds or larger step sizes that produce signatures of creep and hysteresis. Procedures on how to account for those piezo related non-linearities are focus of a forthcoming work . In the (*SM*), we show the breakdown of the linear drift assumptions when the time between two consecutive reference topographies becomes too long.

## Author contributions

J.O. and F.D.N. conceived the project. F.D.N. and J.O. wrote the manuscript. J.O., and F.D.N. analyzed the data. B.Z., D.L., K.H. and F.D.N. measured the data. B.Z. D.L., and K.H. prepared the samples. C.W. and F.O.vR. synthesized the NbSe_2_ samples. F.D.N. supervised the project.

## Declaration of Competing Interest

The authors declare that they have no known competing financial interests or personal relationships that could have appeared to influence the work reported in this paper.

## Data Availability

Data will be made available on request.

## Appendix Supplementary materials

Image, application 1

## Footnotes

1 Sparse sampling, compressive sensing, and compressed sensing are used synonymously.
