# Supplementary material for: Adaptive sparse sampling for quasiparticle interference imaging
Source: MethodsX. 2022 Jul 13;9:101784. doi: 10.1016/j.mex.2022.101784 (PMC9309409; doi:10.1016/j.mex.2022.101784)
Supplement: Supplementary file 1 [file mmc1.pdf]

Supplementary materials and additional information

Sample preparation and spectroscopy

We prepare clean surfaces of Au(111) by repeated cycles of ion bombardment (Ar^+ ions, 1.6×10^{-5} mbar, 25', 4 μA/cm^2) and prolonged annealing at 620 °C. We perform our QPI measurements on terraces exceeding 200 nm. We use an electrochemically etched W-tip that we gently plunge into the Au sample until the apex is atomically sharp, which we verify by scanning across Au step edges. The individual spectra of the main text are recorded using the parallel spectroscopy technique, described earlier(*2*, *6*). High quality single crystals of 2H-$NbSe_2$ were grown by the chemical vapour transport (CVT) method using I_2 as a transport agent. Stoichiometric amounts of Niobium (powder, Alfa Aesar, 325 mesh, 99.99%) and Selenium (shots, ground before use, Alfa Aesar, 99.999%) of a total mass of 0.5 g were sealed in a quartz ampoule (l = 15 cm, ∅l = 9 mm) under vacuum together with 45 mg of I_2 (Fluka, >99%). The ampoule was heated a rate of 180 °C/h in a two zone furnace in which the source zone was fixed at 850 °C and kept at this temperature for 6 days. Eventually, the tube was quenched in cold water. The product was confirmed to be phase pure by powder X-ray diffraction (PXRD). Patterns were collected on an STOE STADI P diffractometer in transmission mode equipped with a Ge-monochromator using Cu $K_{\alpha1}$ radiation and on a Rigaku SmartLab in reflection mode using Cu K_α radiation. A single crystal of $NbSe_2$ is glued together with a cleaving post on top onto the sample holder, transferred without any further heat-treatment into our ultra-high vacuum system, and cleaved in-situ prior to transferring into the cooled scanning tunneling microscope. The spectra of $NbSe_2$ shown below in Figure 4(e) were measured with conventional lock-in spectroscopy.

Time stamp information

For every LDOS location, we measure the timestamp at which the data is recorded, which can be used for drift correction as described in the main text and in our previous work(*1*). Here we mention the two methods we have used to obtain timestamps. The more convenient is to use an event-logger (Swabian Instruments, TimeTagger Ultra), which directly provides a unique timestamp for every measurement location. A more accessible alternative and equally suitable consists in digitizing a periodic sawtooth waveform from an arbitrary waveform generator for time-information. The period should capture several measurement locations to ensure sufficient constraint as to the period number of the sawtooth waveform train. The time-information can then be calculated from the measured voltage level and the period number.

Sparse recovery and measurement matrix

The measured conductance values are coupled to the corresponding locations *k* with example paths that can be found in Ref.(*16*). The general task of the sparse recovery in this case is solving the basis pursuit denoise problem given as $min\ ||x||_1\ s.t.\ ||Ax - y||_2\ \leq \sigma$ where $A\ \epsilon\ \mathbb{C}^{qN\times N}$ is the measurement matrix, $x\ \epsilon\ \mathbb{C}^{N\times 1}$ is the solution to be reconstructed (in its sparse domain, i.e. (complex) Fourier space) and $y\ \epsilon\ \mathbb{R}^{qN\times 1}$ includes the measured data in real space. $\sigma\ \epsilon\ \mathbb{R}$ is a small error threshold that we set in terms of the standard deviation of the measured signal y. We construct the implicit measurement matrix $A = \Omega\Psi$ as a product of a restriction operator $\Omega\ \epsilon\ \mathbb{R}^{qN\times N}$ and a 2D normalized fast Fourier transform operator $\Psi\ \epsilon\ \mathbb{C}^{qN\times N}$. Ω is built such that the row *i* has a single "1" in *i*-th column *k(i)* and thus selects the entries listed in *k* from an input vector of length *N*. These operators are provided within the scope of the SPARCO toolbox(*17*). The measurement matrix A and the measurement vector y are then fed into the sparse recovery algorithm SPGL1(*12*, *13*) along with the error threshold σ. By consecutively increasing the number of measurements, the matrix A will have a higher number of rows, which means that more equations are used, leading to a more faithful reconstruction.

Relative mean absolute error (MAE) as feedback for reconstruction quality

We calculate the relative mean absolute error (MAE) of an image (in our case this is the reconstructed QPI image) with *n* rows and *m* columns as $\frac{1}{nm}\sum_{i=1}^{n}\sum_{j=1}^{m}\left\|\hat{X}_{ij} - X_{ij}\right\|_1/(X_{ij} + \varepsilon)$ where $\hat{X}_{ij}$ and X_{ij} represent the pixel values in the *i*-th row and *j*-th column of the most recent and the previous cumulative ASS snapshots and $\varepsilon > 0$ is a small constant for numerical stability. A drop in the relative MAE over consecutive ASS iterations indicates a gradually approaching ground truth, with smaller gain in QPI quality per iteration.

Limitations of linear drift correction

In our previous work we introduced a linear drift correction procedure(*1*). The assumption of linear drift might not be adequate when there are larger thermal gradients in the system or when the duration between reference topographies is too long. In those situations, our linear drift correction would not improve the spatial relationship between individual measurements. An example in

which our linear drift correction of nonlinear drift worsened the attribution of high-momentum states in $NbSe_2$ is shown in Figure . Panels (a) and (b) show the reconstructed quasiparticle interference patterns of $NbSe_2$ at -100 meV that we obtained on a 50×50 nm^2 region using 6'000 adaptively sampled points, corresponding to two individual TSP paths of 3'000 points each. We recorded the spectra using conventional dI/dV spectroscopy taking 484 ms per spectrum. The grid resolution is 512×512 pixel. Before measuring the spectrum, we wait about 4.5 seconds to settle piezo-electric creep from the previous move to see the impact of thermal drift more clearly.

The main feature that is reconstructed in our ASS sparse recovery are the Bragg peaks forming a hexagonal pattern. Figure (b) shows the energy resolved momenta of $NbSe_2$ after applying linear drift correction. From the zoomed inset we see that the correction did not improve the sharpness of the Bragg peak and no dispersing electron states appear either. The absence of the dispersive electron states is attributed to the low-sampling rate. As mentioned in the main text, we use interleaved dI/dV scans on a fully sampled sub-grid to extract the total drift distances as shown in Figure (c), which separates the ASS segments by 4.5 hours. We then utilize the simultaneously measured time stamps to account for an assumed linear drift to determine the actual locations of the measurements via post-processing. We observe that the applied linear drift correction leads to a worse reconstruction than without any correction as the region around the Bragg peak becomes more smeared. This indicates that our assumption of a purely linear drift does not apply, which we attribute to the hourlong delay between the two ASS segments, as seen by the provided time-stamps shown in Figure (c).

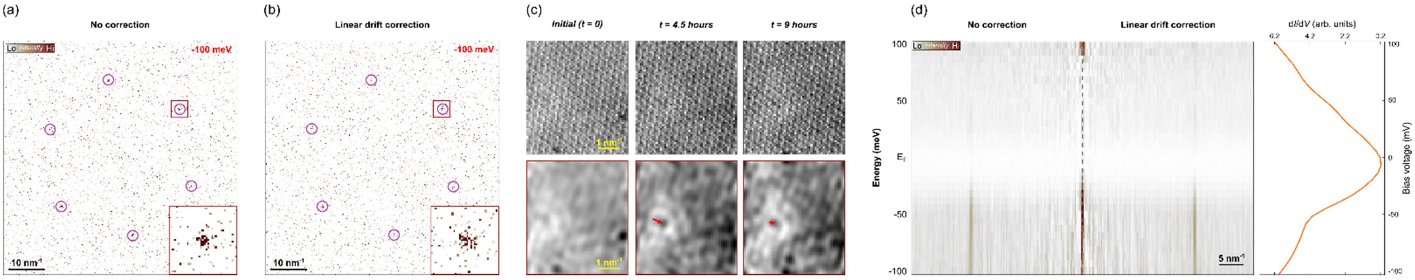

Figure 4: ***Adaptive sparse sampling applied to $NbSe_2$ illustrating the limit of linear drift correction. (a)*** *QPI pattern at -100 meV using two adaptively sampled paths consisting of 3'000 measurement points each. No correction has been applied to the locations of the measurement points.* ***(b)*** *Same as (a) but with a linear drift correction applied that we measured from topographic reference measurements in (c). The Bragg peaks appear more smeared, indicating the detrimental effect of nonlinear drift.* ***(c)*** *Topographic scans (top row) and low pass filtered topographies (bottom row) that we used to determine the drift vectors (in red) for the linear drift correction in (b). The corresponding time tag of the acquisition of each topographic scan is given on top.* ***(d)*** *Energy dependent momentum relation of $NbSe_2$ obtained using a 360-degree azimuthal average, showing the main Bragg peaks but no actual dispersive features, reminiscent of too low sampling rate to ensure their successful reconstruction. The average spectrum of $NbSe_2$ is shown for reference to the right.*

We expect a deviation from a truly linear thermal drift to have less influence on long-range real space features corresponding to low-momentum states in the scattering space as it is the case for Au(111). To investigate this statement, we show in Figure 5 a QPI simulation that we deliberately corrupt by drift. We study what impact our knowledge of the actual drift vectors or the presence of nonlinear drift have on the quality of the QPI pattern when we apply our linear drift correction. We simulate a Shockley surface state with underlying hexagonal atomic lattice as shown in Figure 5(a). In an adaptive sparse sampling fashion, we measure three times 1’000 unique points from a 256×256 pixel grid. After each iteration we shift the underlying ASS scan frame by a small random vector that is proportionally applied to the individual measurements, emulating the effect of a time-dependent thermal drift of the sample. The top panel in Figure 5(a) shows the reconstruction under the correct assumptions of purely linear drift and exact knowledge of the drift vectors. This corresponds to the best result that can be obtained. The lower two panels in Figure 5(a) illustrate what happens if we improperly measure the drift vector and are perturbed by linear drift or if we properly measure the drift vector but the measurements are perturbed by nonlinear drift. The QPI is most impacted in the lowest panel while the middle still shows a fair overall reconstruction. These singular examples are generalized in Figure 5(b)-(d), where we show simulations for different combinations of linear, nonlinear, and improperly measured drift vectors that we quantify using the multiscale structural similarity (MS-SSIM) (*18*) between the reconstructed output and the ground truth (Figure 5(a) top panel). A value of 1 for the MS-SSIM means identical images whereas lower values indicate poorer agreement between the recovered QPI and the ground truth. Our simulations show the phase space for good correction (dark blue regions) becomes small when nonlinear drift is coupled with improperly measured drift. These plots also emphasize the value of operating in the linear drift regime, which can be achieved by frequent interleaving of reference topographies or using fast single point spectroscopies.

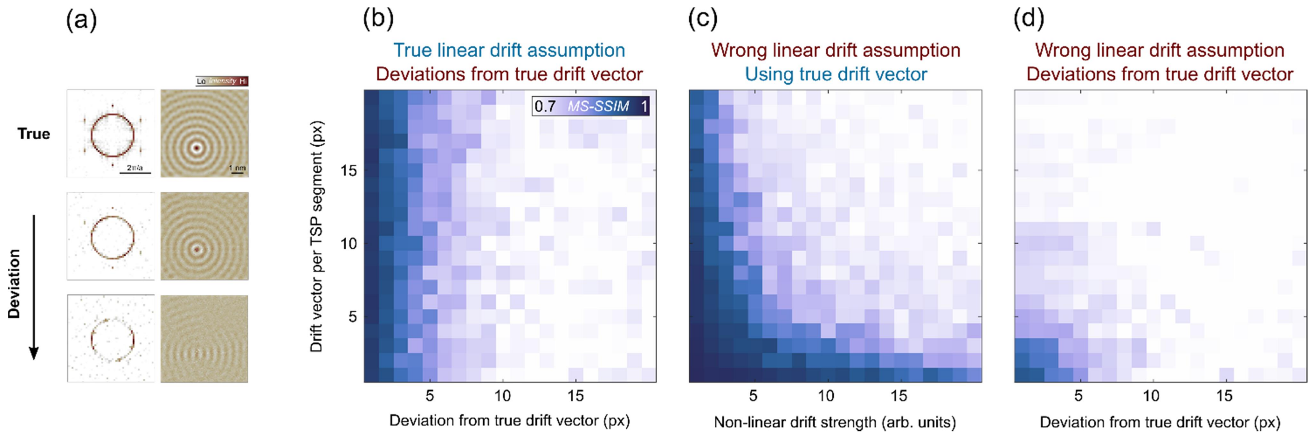

Figure 5: ***Limit of linear drift correction due to wrong drift vector determination or nonlinear drift. (a)*** *Reconstruction of a simulated surface state with linear drift and exact drift-vector determination between each TSP segment consisting of 3x 1’000 points on a 256×256 pixel grid (top panel). The linear drift correction gets worse when the drift vector of a linear drift is improperly measured (middle panel) and encountering nonlinear drift while applying a linear drift correction leads to a loss of QPI features. While the Bragg peaks vanish very early, the surface state is more robust to drift due to its overall shorter scattering wavevectors (larger real space modulations).* ***(b)*** *Averaged simulation over five runs, assuming linear drift but improperly measuring drift vector between each TSP segment (both x and y direction), evaluated using the multiscale structural similarity (MS-SSIM) between the reconstructed surface state and the ground truth. We notice a continuously decreasing quality of the reconstruction with growing error in the drift-vector determination.* ***(c)*** *Correctly measuring the drift vector, correcting for linear drift, but encountering nonlinear drift between each TSP segment. The nonlinear drift is modeled using a quadratic Bézier curve. More nonlinear drift results in a more rapid degradation of the reconstructed QPI pattern.* ***(d)*** *Combination of (b) and (c), that is, erroneously measuring the drift vector and encountering nonlinear drift. The quality of the reconstruction worsens rapidly already for small errors in drift vector assignment and nonlinear drift.*

References

16. J. Oppliger, F. D. Natterer, Measurement Matrix and Path Examples for Adaptive Sparse Sampling for Quasiparticle Interference Imaging (2022), , doi:10.5281/zenodo.6792503.

17. Ewout van den Berg, Michael P. Friedlander, Sparco/ChangeLog at master · MPF-Optimization-Laboratory/Sparco. *GitHub*, (available at https://github.com/MPF-Optimization-Laboratory/Sparco).

18. Z. Wang, E. P. Simoncelli, A. C. Bovik, in *The Thrity-Seventh Asilomar Conference on Signals, Systems Computers* (2003), vol. 2, pp. 1398-1402 Vol.2.
